# Cannabinoid treatment for autism: a proof-of-concept randomized trial

**DOI:** 10.1186/s13229-021-00420-2

**Published:** 2021-02-03

**Authors:** Adi Aran, Moria Harel, Hanoch Cassuto, Lola Polyansky, Aviad Schnapp, Nadia Wattad, Dorit Shmueli, Daphna Golan, F. Xavier Castellanos

**Affiliations:** 1 Neuropediatric Unit, Shaare Zedek Medical Center, 12 Bayit Street, 91031 Jerusalem, Israel; 2 Child Development Centers, Clalit Health Services, Tel Aviv-Yafo, Israel; 3 Child Development Centers, Maccabi Health Services, Jerusalem, Israel; 4 Department of Child and Adolescent Psychiatry, NYU Grossman School of Medicine, New York, NY USA

**Keywords:** Autism spectrum disorder, Cannabinoids, Cannabidiol, Tetrahydrocannabinol, Clinical trials randomized controlled, Neuropsychology, Behavior, Child psychiatry, Developmental disorders, Entourage effect

## Abstract

**Background:**

Endocannabinoid dysfunction in animal models of autism spectrum disorder (ASD) and accumulating, albeit anecdotal, evidence for efficacy in humans motivated this placebo-controlled double-blind comparison of two oral cannabinoid solutions in 150 participants (age 5–21 years) with ASD.

**Methods:**

We tested (1) BOL-DP-O-01-W, a whole-plant cannabis extract containing cannabidiol and Δ9-tetrahydrocannabinol at a 20:1 ratio and (2) BOL-DP-O-01, purified cannabidiol and Δ9-tetrahydrocannabinol at the same ratio. Participants (*N* = 150) received either placebo or cannabinoids for 12-weeks (testing efficacy) followed by a 4-week washout and predetermined cross-over for another 12 weeks to further assess tolerability.

Registered primary efficacy outcome measures were improvement in behavioral problems (differences between whole-plant extract and placebo) on the Home Situation Questionnaire-ASD (HSQ-ASD) and the Clinical Global Impression-Improvement scale with disruptive behavior anchor points (CGI-I). Secondary measures were Social Responsiveness Scale (SRS-2) and Autism Parenting Stress Index (APSI).

**Results:**

Changes in Total Scores of HSQ-ASD (primary-outcome) and APSI (secondary-outcome) did not differ among groups. Disruptive behavior on the CGI-I (co-primary outcome) was either much or very much improved in 49% on whole-plant extract (*n* = 45) versus 21% on placebo (*n* = 47; *p* = 0.005). Median SRS Total Score (secondary-outcome) improved by 14.9 on whole-plant extract (*n* = 34) versus 3.6 points after placebo (*n* = 36); *p* = 0.009). There were no treatment-related serious adverse events. Common adverse events included somnolence and decreased appetite, reported for 28% and 25% on whole-plant extract, respectively (*n* = 95); 23% and 21% on pure-cannabinoids (*n* = 93), and 8% and 15% on placebo (*n* = 94).

Limitations

Lack of pharmacokinetic data and a wide range of ages and functional levels among participants warrant caution when interpreting the results.

**Conclusions:**

This interventional study provides evidence that BOL-DP-O-01-W and BOL-DP-O-01, administrated for 3 months, are well tolerated. Evidence for efficacy of these interventions are mixed and insufficient. Further testing of cannabinoids in ASD is recommended.

*Trial registration* ClinicalTrials.gov: NCT02956226. Registered 06 November 2016, https://clinicaltrials.gov/ct2/show/NCT02956226

## Background

There is no established pharmacological treatment for the core symptoms of autism spectrum disorder (ASD), persistent deficits in social communication, and repetitive, restrictive patterns of behavior [1]; the efficacy and tolerability of pharmacotherapies addressing comorbid disruptive behaviors are relatively low [2].

Consumption of cannabis is reported to enhance interpersonal communication [3] and decrease hostile feelings [4]. The main components of the cannabis plant (phytocannabinoids) are Δ9-tetrahydrocannabinol (THC) and cannabidiol (CBD). THC activates the type-1 cannabinoid receptor (CB_1_R) in the brain; it is psychoactive and can lead to anxiety and psychosis [5]. CBD, on the other hand, is an allosteric modulator of the CB_1_R and might decrease the effects of CB_1_R agonists such as THC. It is not psychoactive and has a relatively high toxicity threshold [5]. While THC consumption, especially at a young age, can lead to addiction, cognitive decline, motivational loss, and psychosis, co-consumption of CBD might reduce these risks [6].

CBD also appears to have anxiolytic, antipsychotic, antiepileptic, and neuroprotective properties that may be mediated through receptors such as serotonin 5-HT_1A_, glycine α3 and α1, TRPV1, GPR55, GABA_A_, and PPARγ, and by inhibiting adenosine reuptake [7–11]. A single oral administration of 600 mg CBD to 34 men (17 neurotypicals and 17 with ASD) increased prefrontal GABA activity in neurotypicals and decreased GABA activity in those with ASD [12].

Epidiolex is a cannabis-derived pure CBD compound which was approved by the U.S. FDA in 2018 for the treatment of two severe forms of epilepsy [13]. This may be relevant for patients with ASD, as 10–30% also have epilepsy, and several pathophysiological pathways are implicated in both disorders [11, 14].

The endocannabinoid system is a cell-signaling system composed of the cannabinoid receptors, their endogenous ligands *(endocannabinoids, mainly* anandamide and 2-AG), transporters, and enzymes which produce and degrade the endocannabinoids [15].

Studies in animal models suggest a reduced endocannabinoid tone in ASD [16–19]. Stimulation of the endocannabinoid system [16–19] and administration of CBD [17] have improved social deficits in some models. Additionally, children with ASD have been found to have lower peripheral endocannabinoid levels [20, 21].

These preclinical data and case-series, reporting treatment with artisanal CBD-rich, cannabis strains [22–26] have triggered widespread use of various cannabis strains in children with ASD, despite a lack of controlled studies. Furthermore, the cannabis plant contains a wide range of minor cannabinoids, terpenes, and flavonoids which differ by strain. These components have also been reported to impact human behaviour [27, 28]. Various combinations of these components have been proposed to have a synergistic pharmacological effect ('the entourage effect') [29]. Whether presumed effects of cannabis in ASD should be attributed to CBD or THC, or whether minor cannabinoids, terpenes, and flavonoids also contribute therapeutically remains unclear. Accordingly, we performed a proof-of-concept, placebo-controlled trial of whole-plant extract and pure cannabinoids in children and adolescents with ASD. We hypothesized that whole-plant extract, per the entourage effect, would be more effective than placebo for disruptive behaviors; assessing this hypothesis was our primary objective. A secondary objective was to assess the efficacy of pure cannabinoids which are more standardized and repeatable than whole-plant extracts and hence more suitable for pharmacotherapy.

## Methods

### Standard protocol approvals, registrations, and patient consents

NCT02956226 was approved by the Institutional Review Board at Shaare Zedek Medical Center and the Israeli Ministry of Health prior to participant enrollment. Participants’ parents provided written informed consent and written assent was obtained from participants when appropriate.

### Study design

This proof-of-concept, randomized, double-blind, placebo-controlled trial was conducted in a single referral center—Shaare Zedek Medical Center, Jerusalem, Israel. Eligible participants were children and adolescents (5–21 years old) with an ASD diagnosis per DSM-5 criteria, confirmed by Autism Diagnostic Observation Schedule (ADOS-2), and moderate or greater behavioral problems (rating ≥ 4) on the Clinical Global Impression (CGI)-Severity scale (Table 1). Anchoring instructions (provided in the Additional file 1) were used so that the CGI-S would quantify behavioral difficulties rather than overall ASD severity.

**Table 1:** Inclusion and exclusion criteria for study participation

Inclusion criteria	1. Male or female outpatients aged 5–21 years old^a^ 2. Diagnosis of ASD according to Diagnostic and Statistical Manual of Mental Disorders [Fifth Edition; DSM-5] 3. Moderate or greater behavioral problems as measured by a Clinical Global Impression Scale—Severity (CGI-S) score of 4 or higher at screening^b^ 4. Involvement of a parent or caregiver able to consistently complete assessments throughout the study
Exclusion criteria	1. Lifetime history of psychotic disorder 2. Current or former treatment with cannabinoids 3. A medical condition (such as heart, liver, renal or hematological disorder) that impacts the subject's ability to participate in the study or makes the subject predisposed to severe adverse events 4. Subjects who have had changes in pharmacological, educational, or behavioral treatments for 4 weeks prior to randomization or planned changes in existing interventions for the duration of the trial

^a^In Israel, special education programs for individuals with ASD and neuropediatric clinics continue to follow patients with ASD until they are 21 years old

^b^To assign CGI-S scores, structured criteria were used to rate behavioral difficulties on the CGI-S, rather than overall ASD severity

Participants were randomly assigned (1:1:1 ratio) to 1 of 3 treatments for 12-weeks. Treatments were: (1) oral placebo, (2) whole-plant cannabis extract containing CBD and THC at a 20:1 ratio, and (3) pure CBD and pure THC at the same ratio and concentration. Randomization and blinding processes are described in the Additional file 1.

The primary objective was to evaluate whether whole-plant cannabis extract would induce a significant improvement in behavioral assessments compared to placebo. We used the same CBD: THC ratio as in previous open-label case series [22–24]. We did not use a ‘CBD only’ arm in this initial study, as we hypothesized that the CBD-THC combination would be more efficacious because of direct effects of THC on the endocannabinoid system.

**Fig. 1:**
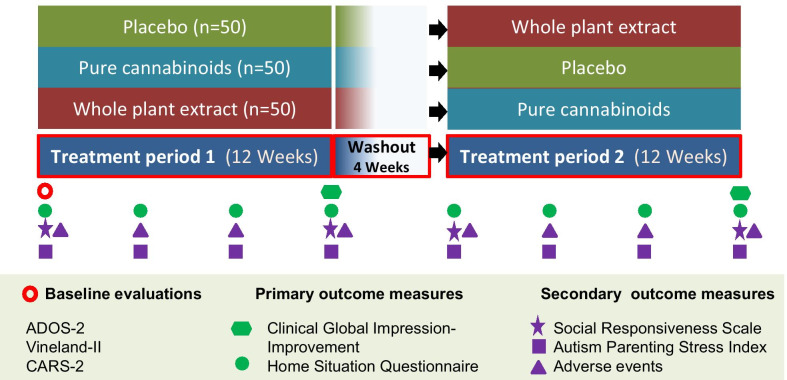
Study design

For ethical reasons, we used a crossover design in which all participants would receive cannabinoids at least once: after 12-weeks of treatment (‘Period-1’) and a 4-week washout period, participants crossed-over to a predetermined second 12-week treatment (‘Period-2’; Fig. 1). The cross-over design was intended to allow within-participant analyses, comparing the two treatments that each participant received. As we had noted a substantial improvement in our open observational study with whole-plant extract [22], we ordered treatments a priori to minimize the likelihood of substantial improvement of severe disruptive behaviors in the first period and deterioration in the second period. As we hypothesized that whole-plant extract would be more effective than pure cannabinoids, we excluded the sequence of whole-plant extract followed by placebo.

Preliminary analyses revealed a treatment order effect: change from baseline was greater in the first period than in the second, suggesting a greater initial placebo effect. As a treatment order effect impairs the validity of within-participant analyses, we decided to evaluate between-group efficacy only during the first period. Data from both periods were examined for safety and tolerability. For transparency, we present within-participant analyses and between-participant analyses of period-2 (Additional file 1).

### Intervention

Cannabis plants (Topaz strain; BOL Pharma, Israel) were subjected to CO_2_ extraction. The extract was either immediately dissolved in olive oil (BOL-DP-O-01-W) or underwent further purification to 99% pure CBD and then was dissolved in olive oil (BOL-DP-O-01). The final concentrations of CBD and THC in both solutions were 167 mg/ml CBD and 8.35 mg/ml THC. Flavorings were added to all three solutions to make taste and scent uniform.

In each treatment period, starting dose was 1 mg/kg/d CBD (and 0.05 mg/kg/d THC). The dose was increased by 1 mg/kg/d CBD (and 0.05 mg/kg/d THC) every other day up to 10 mg/kg body weight per day CBD (and 0.5 mg/kg/d THC) for children weighing 20–40 kg or 7.5 mg/kg/d CBD (and 0.375 mg/kg/d THC) for weight > 40 kg (to a maximum of 420 mg CBD and 21 mg THC per day) divided into 3 daily doses. Treatments were given orally (sublingual whenever possible) as an add-on to any ongoing stable medication. At the end of each treatment period, the study treatment was gradually decreased over 2 weeks.

### Baseline evaluations

Baseline assessments included: ADOS-2 [30], a standardized assessment of communication, social interaction, play, and imaginary use of materials; Vineland Adaptive Behavior Scales (VABS) [31], a caregiver interview assessing Communication, Socialization, and Daily Living Skills; and Childhood Autism Rating Scale-Second edition (CARS2-ST) [32], a quantitative measure of direct behavior observation.

### Outcomes

*Primary outcomes:* We designated two co-primary outcome measures to assess ASD associated disruptive behaviors: Home Situations Questionnaire-ASD (HSQ-ASD) and CGI-Improvement (CGI-I) targeting behavioral problems.

*HSQ-ASD *[33] is a 24-item parent-rated measure of noncompliant behavior in children with ASD. The scale yields per-item mean scores of 0 to 9 (higher is worse) [33].

*CGI-I *[34] was used to measure improvement in disruptive behaviors from baseline by incorporating anchoring instructions related to behavioral difficulties (Anchors appear in the Additional file 1). As in the standard CGI-I, scores ranged from 1 (very much improved) through 4 (unchanged) to 7 (very much worse). Scores of 1 or 2 (much improved) were defined as a positive response; all others indicated a negative response [34]. CGI-I was assessed at the end of each treatment period. The same clinician (AA) assessed and rated the CGI-S and CGI-I of all participants.

*Secondary outcomes* included the Social Responsiveness Scale-2nd edition (SRS-2), the Autism Parenting Stress Index (APSI), and adverse events.

*SRS-2*: [35] this 65-item, caregiver questionnaire quantifies autism symptom severity (total scores range from 0 to 195; higher is worse).

*APSI: *[36] this 13-item parent-rated measure assesses parenting stress in three categories: core social disability, difficult-to-manage behavior, and physical issues.

*Adverse events* were assessed using a modified Liverpool Adverse Events Profile (LAEP) including the 19 original LAEP [37] items plus 15 items covering all significant adverse effects of CBD and THC reported in prior pediatric studies.

### Statistical analyses

The primary aim of this study was to test the superiority of whole-plant-extract over placebo in treating ASD associated behavioral problems, using the HSQ-ASD and the CGI-I for disruptive behaviors. The comparison between pure-cannabinoids and placebo was registered as a secondary outcome. Sample size calculation was based on an effect size of *f* = 0.67 (in total HSQ-ASD score) [38] and standard deviation of 3 points in the within-participant difference between placebo and whole-plant extract conditions. To achieve 80% power with 2.5% alpha (adjusted for two co-primary endpoints) requires a sample of 43 patients per group. To account for attrition, an additional 15% were enrolled. A total of 50 participants per arm was set to test primary study endpoints. Analyses were performed using JMP version 14 (SAS Institute, Cary, NC, USA). All P values were two-sided. Specific statistical tests used and corrections applied for multiple comparisons are indicated in figure/table legends.

For details on the cannabinoid preparations, randomization process, important changes to methods after trial commencement, anchoring instructions for rating the CGI-S and CGI-I, and the CONSORT checklist, see Additional file 2.

## Results

Between 11 January 2017 and 12 April 2018, 150 children and adolescents (mean age 11.8 ± 4.1 years, median 11.25, range 5.1–20.8; 80% boys) entered the trial. ASD symptoms were ‘severe’ in 78.7% per ADOS-2 (Comparison Score = 8–10) [30] and adaptive level was ‘low’ (Standard Score ≤ 70) in 88% per Vineland Behavior Scales [31].

**Fig. 2:**
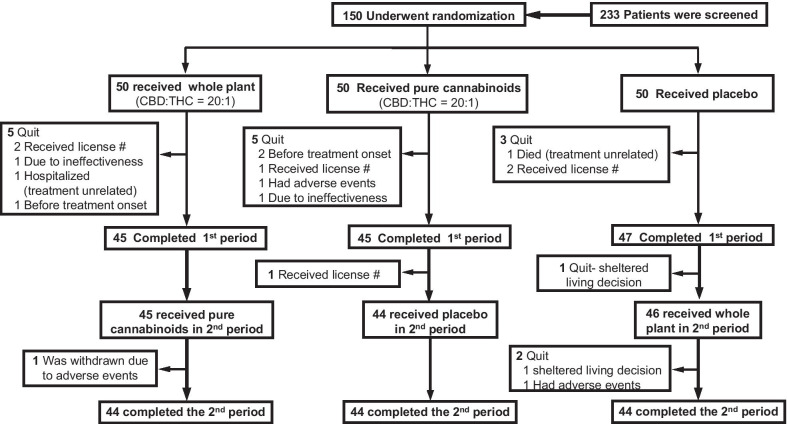
Trial profile: screening, randomization and treatment periods

Screening, randomization and attrition are shown in Fig. 2 and participant characteristics are provided in Table 2. Fifty participants were randomly assigned to each of the 3 treatments in Period-1 and 44 per group completed the study (12% overall attrition).

**Table 2:** Participant characteristics

	All	Placebo in 1st period; whole- plant in the 2nd	Pure cannabinoids in 1st period; placebo in the 2nd	Whole-plant in 1st period; pure cannabinoids in 2nd	*P*-value^a^
Age: mean ± SD [median, range]	11.8 ± 4.1 [11.3, 5.1–20.8]	11.7 ± 3.8 [10.7, 5.8–20]	11.6 ± 4.3 [10.3, 5.1–20.4]	12.1 ± 4.3 [12.6, 5.1–20.8]	0.79
Sex (% girls)	20%	16%	16%	28%	0.22
ADOS-2 Total Score mean ± SD [median, range]	21.8 ± 6.0 [23, 7–32]	22.1 ± 6.5 [23.5, 7–32]	22.5 ± 5.8 [24, 11–32]	20.9 ± 5.8 [21, 9–30]	0.41
VABS Standard Score mean ± SD [median, range]	52.3 ± 14.5 [51, 20–102]	52.0 ± 15.0 [49, 26–102]	52.4 ± 15.2 [54, 25–89]	52.3 ± 13.6 [52, 20–78]	0.27
CARS Total Score mean ± SD [median, range]	45.4 ± 8.4 [47.5, 29.5–59]	46.0 ± 8.5 [47.5, 30.5–59]	45.5 ± 8.9 [48.5, 29.5–57.5]	44.6 ± 7.8 [46.5, 31–56.5]	0.55
CGI-S maladaptive behavior mean ± SD [median, range]	5.6 ± 0.7 [6, 4–7]	5.5 ± 0.7 [6, 4–7]	5.6 ± 0.7 [6, 4–7]	5.6 ± 0.7 [6, 4–7]	0.78
HSQ Total Score (baseline) mean ± SD [median, range]	3.5 ± 1.7 [3.3, 0.3–8.5]	3.7 ± 1.5 [3.7, 0.7–6.0]	3.2 ± 1.5 [3.1, 0.7–6.6]	3.7 ± 2.1 [3.6, 0.3–8.5]	0.33
SRS-2 Total Score (baseline) mean ± SD [median, range]	119 ± 27 [121, 53–180]	122 ± 23 [124, 53–159]	118 ± 31 [118, 64–178]	117 ± 27 [117, 66–180]	0.37
APSI Total Score (baseline) mean ± SD [median, range]	27.1 ± 10.4 [26, 7–54]	28.3 ± 10.3 [27, 11–50]	25.8 ± 10.4 [25, 8–54]	27.4 ± 10.7 [25, 7–48]	0.67
BMI (baseline) mean ± SD [median, range]	20.8 ± 5.7 [19.0, 12.3–39.6]	20.5 ± 5.2 [19.1, 12.8–34]	20.5 ± 6.0 [19.1, 12.3–39.6]	21.3 ± 6.1 [19.0, 13.9–39.6]	0.67
Epilepsy	9%	8%	8%	10%	0.92
*Concomitant medications*					
Any medication	72%	72%	68%	76%	0.67
Antipsychotics	54%	58%	44%	60%	0.22
SSRIs	15%	12%	16%	16%	0.80
Antiepileptics (also given as mood stabilizers)	12%	12%	12%	12%	1.0
Stimulants	12%	8%	22%	6%	0.033
Benzodiazepines	7%	2%	8%	10%	0.19
Alpha-2 agonists	4%	4%	2%	6%	0.58

*ADOS-2* Autism Diagnostic Observation Schedule-2nd edition, (Modules 1, 2 and 3 were used for 55%, 17%, and 28% of the participants, respectively, without significant differences among the 3 study arms); *VABS* Vineland Adaptive Behavior Scales; *CARS* Childhood Autism Rating Scale; *CGI-S* Clinical Global Impression–Severity [5 = markedly ill, 6 = severely ill, 7 = among the most extremely ill patients; all referencing disruptive behaviors]; *HSQ* Home Situations Questionnaire; *SRS-2* Social Responsiveness Scale-2nd edition; *APSI* Autism Parenting Stress Index; *SSRIs* Selective serotonin reuptake inhibitors

^a^Categorical parameters (sex, epilepsy and medications) were compared using likelihood ratio chi-square tests. Continuous parameters were compared using the Kruskal–Wallis test if data distribution was non-normal but similar across groups (BMI) and using median tests if data distribution was non-normal and different across groups (age, assessment scores)

### Safety and tolerability of cannabinoid treatment with BOL-DP-O-01-W (whole-plant extract) and BOL-DP-O-01 (pure cannabinoids)

Adverse events (AEs) were reported whenever they occurred, and caregivers were proactively asked about them at each study visit, and every 4 weeks using a structured questionnaire. AEs were documented whether considered related to study treatments or not. Reports of new adverse events or worsening of previously reported events were rated mild (present, but not problematic), moderate (problematic and leading to study drug dose decrease), or severe (posing a problem requiring medical intervention). Serious AEs were possibly life-threatening events or any requiring hospitalization. Overall, 95 participants received a whole-plant extract, 93 received pure cannabinoids, and 94 received a placebo.

There were no treatment-related severe or serious AEs. Six participants had an unrelated serious event (Additional file 1: Table S1). Overall, mild AEs were not significantly more frequent during cannabinoid treatment (mild AEs were reported 383, 388, and 353 times, in 89, 79, and 78 participants during treatment with whole-plant extract, pure cannabinoids, and placebo, respectively). Moderate AEs were reported 80, 78, and 57 times, in 44, 45, and 26 participants during treatment with whole-plant extract, pure cannabinoids, and placebo, respectively. AEs that were more common during cannabinoid treatment are presented in Table 3. The full list of adverse events and correlations with age, sex, treatment dose, and concomitant medications appears in Additional file 1: Table S2.

**Table 3:** Common adverse events reported during either 12-week treatment period

	Whole-plant extract CBD 5.5 mg/kg/d; n = 95 (%)	Pure cannabinoids CBD 5.5 mg/kg/d; n = 93 (%)	Placebo n = 94 (%)	*P* value (placebo vs cannabinoids)
**Somnolence**	**27**	**24**	**7.5**	** < 0.001**
Mild	20	18.5	7.5	
Moderate	7	5.5	0	
Severe	0	0	0	
**Decreased appetite**	**24**	**22**	**15**	**0.157**
Mild	21	16.5	13	
Moderate	3	5.5	2	
Severe	0	0	0	
**Weight loss**	**12**	**13**	**4**	**0.053**
Mild	9	12	3	
Moderate	3	1	1	
Severe	0	0	0	
**Tiredness**	**25**	**34**	**19**	**0.077**
Mild	21	28.5	18	
Moderate	4	5.5	1	
Severe	0	0	0	
**Euphoria**	**20**	**19**	**13**	**0.201**
Mild	15	16	12	
Moderate	5	3	1	
Severe	0	0	0	
**Anxiety**	**20**	**27**	**14**	**0.084**
Mild	17	25	11	
Moderate	3	2	3	

CBD: cannabidiol (CBD:THC ratio was 20:1 for both cannabinoids tested; the average daily dose per kg was lower than the target dose as many participants weighted over 42 kg and reached the maximal daily dose)

Bold: sum of mild + moderate + severe for each adverse event

### Impact of cannabinoid treatment with BOL-DP-O-01-W (whole-plant extract) and BOL-DP-O-01 (pure cannabinoids) on behavior

**Fig. 3:**
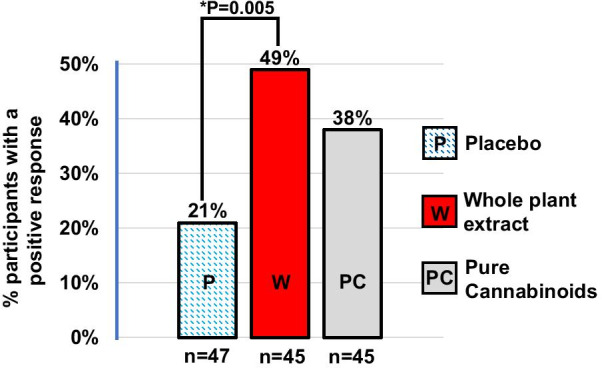
Participants (%) whose behavioral problems either much improved or very much improved on the CGI-I scale following treatment. Response to 12-week treatment using the Clinical Global Impression-Improvement (CGI-I). Positive response in this scale is defined as a rating of ‘much improved' or 'very much improved' [34]*.* Outcome was analyzed using Likelihood ratio chi-square test. *P* value is unadjusted. *Remains significant after Bonferroni-correction for multiple comparisons

The impact of cannabinoid treatment on behavioral problems was assessed using the HSQ-ASD [33], and the CGI-I [34] (co-primary outcome measures). The APSI [36] (secondary outcome measure) also reflects the child’s behavior. HSQ-ASD total scores and APSI total scores did not differ significantly between participants who received cannabinoids and participants who received placebo (Table 4). On the CGI-I, 49% of 45 participants who received whole-plant cannabinoids responded (either much or very much improved) [34] compared with 21% of 47 on placebo (*p* = 0.005, Fig. 3). Of the 45 participants who received pure cannabinoids, 38% responded, which was not significantly higher than placebo (*p* = 0.08).

**Table 4:** Impact of cannabinoid treatment, as reflected by change from baseline to end of treatment period 1 in total scores of HSQ-ASD, SRS-2, and APSI

	Median (range) [*n*]			Pairwise P		
Assessment	Whole-plant extract	Pure cannabinoids	Placebo	Whole-plant versus placebo	Pure C. versus placebo	Whole-plant versus pure C
HSQ-ASD	− 1.1 (− 3.8 to 1.6) [40]	− 0.7 (− 4.4 to 3.8) [42]	− 0.5 (− 3.7 to 2.5) [39]	0.575	0.915	0.508
SRS-2	− 14.9 (− 45 to 15) [34]	− 8.2 (− 69 to 45) [28]	− 3.6 (− 63 to 35) [36]	**0.009**	0.801	0.202
APSI	− 5.4 (− 39 to 13) [38]	− 4.9 (− 19 to 22) [42]	− 1.5 (− 26 to 20) [42]	0.502	0.513	0.991

*HSQ* Home Situations Questionnaire–ASD; *SRS-2* Social Responsiveness Scale-2nd edition; *APSI* Autism Parenting Stress Index

Median tests were used, as distributions were non-normal

*P*-values are based on Mood’s Median Test of each pairwise comparison

None of these 3 measures (HSQ-ASD, CGI-I and APSI) differed significantly between participants who received whole-plant extract versus pure cannabinoids (Table 4).

Second treatment period results are presented in Additional file 1: Table S3 and Additional file 1: Figure S2 for transparency but not further discussed because of a significant order effect.

### Impact of BOL-DP-O-01-W (whole-plant extract) and BOL-DP-O-01 (pure cannabinoids) on Social Responsiveness Scale scores

ASD symptoms (secondary outcome) were assessed with the SRS-2 [35]. Improvement in SRS-2 total score was significantly higher following treatment with whole-plant extract compared with placebo (Table 4). Median total score improved by 3.6 points after placebo (*n* = 36) versus 14.9 on whole-plant extract (*n* = 34; *p* = 0.009) and 8.2 on pure cannabinoids (*n* = 28; *p* = 0.80). Results of the second treatment period are presented in Additional file 1: Table S3 and Additional file 1: Figure S3 for transparency.

### Exploratory analyses: impact of BOL-DP-O-01-W (whole-plant extract) and BOL-DP-O-01 (pure cannabinoids) treatment on Body Mass Index (BMI)

Baseline BMIs were equivalent across treatment groups (Table 2). The BMI of participants who received cannabinoids decreased during active treatment [Median {25%, 75%}] by − 0.45 {− 1.15, 0.18} in Period-1 (*n* = 44) and − 0.12 {− 0.77, 0.18} in Period-2 (*n* = 40)] following treatment with whole-plant extract; BMI decreased by − 0.36 {− 1.09, 0.24} in Period-1 (*n* = 44) and − 0.01 {− 0.61, 0.48} in Period-2 (*n* = 43) following treatment with pure cannabinoids. Changes in BMI following cannabinoid treatment (either whole-plant extract or pure cannabinoids) were − 0.36 {− 1.14, 0.2} in Period-1 (*n* = 88) and − 0.01 {− 0.7, 0.38} in Period-2 (*n* = 83). During treatment with placebo, changes in BMI were 0.16 {− 0.25, 0.56} in Period-1 (*n* = 43; *p* < 0.0001 versus cannabinoids) and 0.30, {0, 0.96} in Period-2 (*n* = 43; *p* = 0.002 versus cannabinoids).

**Fig. 4:**
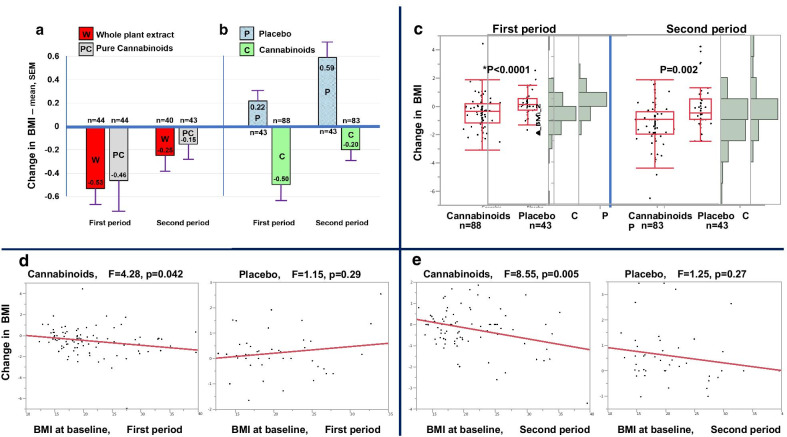
Impact of cannabinoid treatment on BMI. Change in BMI during 12-week treatment with either cannabinoids or placebo. **a** Whole-plant extract versus pure cannabinoids; **b** Cannabinoid treatment (either whole-plant extract or pure cannabinoids) versus placebo; **c** Distribution of data, bars represent 10%, 25%, Median, 75% and 90%, differences between placebo and cannabinoids were analyzed using median test; **d**, **e** Change in BMI as function of baseline level, stratified by treatment (cannabinoids or placebo) and treatment period (**d** first period, **e** second period). Correlations were analyzed using linear regression

Notably, participants with higher BMI at baseline had a more prominent decrease in BMI following cannabinoid treatment [The decrease in BMI was positively correlated with baseline BMI (F = 4.3, *p* = 0.042 in Period-1, F = 8.6, *p* = 0.005 in Period-2)]. Change in BMI following placebo was not significantly correlated with baseline BMI (Fig. 4).

### Exploratory analyses: possible moderators of treatment effects

Additional file 1: Table S4 presents possible moderators of treatment response. Severity of ASD core symptoms at baseline (as assessed by ADOS-2) and concomitant use of medications were not significantly associated with response to either pure cannabinoids or whole-plant extract, on any assessment.

Males were more likely to improve on the HSQ-ASD and SRS-2. Younger children were more likely to improve on the CGI-I and APSI. Participants who had somnolence during cannabinoid treatment were more likely to respond per the CGI-I assessment. However, treatment with the whole-plant extract remained significantly associated with improvement on the CGI-I and SRS-2 after controlling for somnolence and for concomitant use of medications during treatment [Odds Ratio {95% confidence interval} of 6.08 {1.91, 21.82} (*p* = 0.003) and 3.56 {1.31, 10.28} (*p* = 0.015), respectively].

Correlations between treatment dose (per Kg of body weight) and treatment response are presented in Additional file 1: Table S5. The average treatment dose during the first period was 5.7 ± 2.6 mg/kg/d of CBD in the whole-plant extract arm and 5.9 ± 2.7 mg/kg/d of CBD in the pure cannabinoids arm. A higher dose of whole-plant extract correlated with higher behavioral improvement on the CGI-I (*r*_s_ =  − 0.29, *n* = 45, *p* = 0.050). Cannabinoid dose did not correlate significantly with any other endpoints for either whole-plant extract or pure cannabinoids.

### Concomitant medications

Study treatments were added to ongoing behavioral or pharmacological treatments. Planned changes in such treatments or a change in the 4 weeks prior to randomization were exclusionary.

Concomitant medications were taken by 72% of participants (Table 2). Adverse events or response were not significantly associated with concomitant medication use (Additional file 1: Table S2 and S3), except for somnolence which was higher in those on chronic medications (*p* = 0.001).

### Discussion

Currently, there are no established medications for the core autistic symptoms. Risperidone and aripiprazole have been approved by the U.S. Food and Drug Administration (FDA) to treat comorbid irritability [2] but these medications often cause obesity and metabolic syndrome [2, 39].

In this study, we have demonstrated for the first time in a placebo-controlled trial that cannabinoid treatment has the potential to decrease disruptive behaviors associated with ASD, with acceptable tolerability. This is specifically important for the many individuals with ASD who are overweight, as cannabinoid treatment was associated with net weight-loss (Fig. 4) in contrast to the substantial weight gain usually produced by antipsychotics.

Two co-primary outcomes were designated to assess improvement in disruptive behaviors following cannabinoid treatment: a parent questionnaire (HSQ-ASD) and an interview-based clinician assessment (CGI-I).

HSQ-ASD scores did not differ significantly between participants who received cannabinoids and participants who received placebo. However, as our cohort included children and adolescents with a wide range of function levels, many participants had 4 or more items which were not applicable on the HSQ-ASD, limiting sample size on this scale (Table 4).

The clinician assessment was based on a detailed description of the most bothersome behavioral problems at baseline and an extensive interview at the end of each treatment period focused on those problems. Using this patient- and family-centered tool customized for each participant, we found that 49% of participants receiving the whole-plant extract treatment responded versus 21% on placebo (*p* = 0.005).

Intriguingly, one of our secondary outcomes, the SRS-2, provided preliminary evidence that cannabinoid treatment might improve core symptoms of ASD (Table 4). This finding could be of high importance if confirmed in future studies, as studies exploring pharmacological interventions for the ASD core symptoms are scarce.

Although not reportable as evidence of efficacy due to crossover effects, Additional file 1: Figures S2 and S3 show that results in the second treatment period were similar to those in the first.

Other possible implications of this preliminary study for future studies and selected clinical use include feasibility of sublingual administration in children with low adaptive level, and feasibility of a starting dose of 1 mg/kg/d of CBD and a gradual increase over 2–3 weeks to a target of 5–10 mg/kg/d divided into 2–3 daily doses.

The study explored two cannabinoid compounds, differing by the absence of terpenes, flavonoids, and minor cannabinoids in the pure-cannabinoid compound. While additive and even synergistic therapeutic effects of these additional components have been suggested ('entourage' effect) [28, 29], we did not find clear advantages for the whole-plant extract over pure cannabinoids, suggesting that attempts to search for the optimal 'entourage' effect across cannabis strains with the same CBD:THC ratio are likely to be challenging. As previously reported in studies of children with refractory epilepsy [40, 41], we also found relatively high placebo effects, emphasizing the importance of placebo in studies of medical cannabis.

Similar to these studies we also found somnolence to be the most prevalent adverse event but importantly, cannabinoid treatment remained significantly associated with a positive response on the CGI-I and SRS-2 assessments after controlling for somnolence during treatment [Odds ratio of 6.08, *p* = 0.003].

Cannabinoids might affect behavior and communication through several mechanisms. THC activates CB_1_R and has been associated with enhanced social behavior in multiple studies [42, 43]. CBD is a 5-HT_1A_ receptor agonist, which might facilitate anxiolytic effects. Its presumed antipsychotic effect is attributed to partial agonism at dopamine D2 receptors, similar to the antipsychotic action of aripiprazole [44].

### Limitations

Our study had several limitations. Although it was designed as a cross-over study, preliminary analyses revealed a treatment order effect which prevented the use of data from the second treatment period and limited sample size. As this was the first clinical study in the ASD field, we included a wide range of levels of function. Unfortunately, the standardized questionnaires contained many items that were inapplicable for some low-functioning participants, resulting in numerous invalid scores and decreased statistical power on those measures. We did not perform genetic or intelligence quotient evaluations and could not assess the effects of genetic background or cognitive level on treatment response. We did collect data on concomitant medications but were not powered to detect effects on treatment response or on adverse events. We did not obtain data on pharmacokinetics of the interventions and concomitant medications nor tests of liver enzymes and complete blood count, although we detected no clinical evidence of hepatic or hematologic dysfunction.

### Conclusions

Novel pharmacological treatments for the core and comorbid symptoms of ASD are urgently needed. Preclinical studies implicate the endocannabinoid system in the pathophysiology of ASD. In a controlled study of 150 participants, we found that BOL-DP-O-01-W, a whole-plant extract which contains CBD and THC in a 20:1 ratio, improved disruptive behaviors on one of two primary outcome measures and on a secondary outcome, an index of ASD core symptoms, with acceptable adverse events. These data suggest that cannabinoids should be further investigated in ASD.

Future studies should consider recruiting participants within narrower ranges of age and functional levels, assess the long-term tolerability and safety of cannabinoid treatments, and identify target populations within the autism spectrum that might benefit most from these treatments.

## Supplementary Information

**Additional file 1.** Data Supplement.

**Additional file 2.** CONSORT checklist.

## Data Availability

The authors declare that the data supporting study findings are available within the paper and its Additional file. The remaining data are available from the corresponding author upon reasonable request.

## Abbreviations

APSI: Autism Parenting Stress Index
ASD: Autism spectrum disorder
CB_1_R: Type-1 cannabinoid receptor
CBD: Cannabidiol
CGI-S/CGI-I: Clinical Global Impression–Severity/Improvement
HSQ: Home Situations Questionnaire
SRS: Social Responsiveness Scale
THC: Tetrahydrocannabinol
