# Supplementary material for: Cannabinoid treatment for autism: a proof-of-concept randomized trial
Source: Mol Autism. 2021 Feb 3;12:6. doi: 10.1186/s13229-021-00420-2 (PMC7860205; doi:10.1186/s13229-021-00420-2)
Supplement: Supplementary file 1 — Additional file 1. Data Supplement. [file 13229_2021_420_MOESM1_ESM.docx]

Additional file

*Appendix 1: Interventions; Randomization & blinding; and Changes to methods*

*Appendix 2:* Anchoring instructions for rating the CGI-S and CGI-I – Behavioral Problems - ASD

*Supplemental Tables 5,6,7,8,9*

*Supplemental Figures 5,6,7*

**Appendix 1**

**Interventions**

All treatments were given as oil-based extracts for sublingual administration by drops containing 0.034 ml each ([BOL pharma](https://www.bolpharma.com/), Israel, FDA global G.A.P. approved).

Placebo solution was prepared to imitate the flavor and color of the whole-plant extract:

| Material | Amount in % weight | Amount per unit (per ml)  Amount per drop (≈0.04ml) | Function |
| --- | --- | --- | --- |
| Olive oil | 98.89% | 0.9889ml /ml; 0.0395ml/drop | Solvent |
| Oleoresin oregano | 1% | 0.01ml / ml; 0.004ml/drop | Color and flavor masking |
| Raspberry o.s. | 0.03% | 0.003ml/ ml; 0.00012 ml/drop | Flavor enhancer |
| Paprika color | 0.08% | 0.008ml/ml; 0.00032ml/drop | Color |

Pure cannabinoids were mixed with the placebo solution in the following ratios:

| Material | Amount (W/W%) | Amount per unit (per ml)  Amount per drop (≈0.04ml) | Function |
| --- | --- | --- | --- |
| CBD | 18.2% (182 mg/gram) | 167 mg/ml; 6.7mg/drop | API |
| THC | 0.91% (9.1 mg/gram) | 8.3 mg/ml; 0.332mg /drop | API |
| Placebo solution | 80.89% (Completion to 100% formulation) |  | Solvent |
|  |  |  |  |

Whole-plant extract was mixed with the placebo solution in the following ratios:

| Material | Amount (W/W%) | Amount per unit (per ml)  Amount per drop (≈0.04ml) | Function |
| --- | --- | --- | --- |
| CBD | 18.2% (182 mg/gram) | 167 mg/ml; 6.7mg/drop | WP |
| THC | 0.91% (9.1 mg/gram) | 8.3 mg/ml; 0.332mg /drop | WP |
| Placebo solution | 80.89% (Completion to 100% formulation) |  | Solvent |

The products were supplied to Shaare Zedek Medical Center in 6 batches (over 22 months of study duration). Certificate of Analysis from a certified, independent laboratory (Izun Pharma, Jerusalem, Israel) was supplied with each batch. Stability of the products was approved by the Israeli Ministry of Health to be 18 months at room temperature. Specification sheets of the products are available upon reasonable request. Surprisingly, given the low functioning of many participants and prior reports of reluctance to take other medications, only two participants were reluctant to take the intervention formulations.

**Randomization and blinding**

Eligible subjects were assigned an enrollment number in sequential order beginning with 01. Allocation to treatment arm was based on a randomization list. Randomization scheme was generated by BioStats Statistical Consulting Ltd. (an external biostatistical consulting firm). The blocked randomization was performed using SAS statistical software V9.4 via the plan procedure with a fixed block size.

The randomization scheme and lists were sent to the center for both periods of the study. The randomization list included sequential numbers and an assigned 3-letter code (several codes were assigned for each of the 3 treatment options). Randomization was kept at 1:1:1 ratio. The randomization list was encrypted by password and was sent prior to first dosing to the principal investigator.

The study whole-plant extract, the pure cannabinoids as well as placebo were packed in similar bottles and had similar color, taste and smell characteristics. The treatment products and placebo were both dissolved in olive oil. Addition of paprika color and oleoresin oregano to the placebo solution disguised the color completely. Addition of oleoresin oregano at high concentration was applied to mask the tastes of CBD and THC and raspberry flavor was added to mask the aroma (confirmed by a group of experienced tasters).

The code key was kept by BioStats Statistical Consulting Ltd. until study end. Neither the principal investigator nor any other team member or individual had access to the codes until study end. No unblinding occurred during the study.

**Important changes to methods after trial commencement**

The following changes were made after trial commencement in January 2017.*

| Change | Date | Reason |
| --- | --- | --- |
| Eligibility age was narrowed: 5-21 years instead of 5 – 29 years | June 2017 | The recruitment rate of children under 21 years was sufficient and there was no need to use the wider age range (which was planned to be used only in a case of insufficient recruitment) |
| Anticipated enrollment was increased from 120 to 150 | January 2018 | Concerns about attrition (medical cannabis became legally available for ASD in Israel) and a revised statistical consultation |
| CBCL (previously a secondary outcome measure) was excluded | April 2018 | Many items in this instrument (which was not designed for ASD) are not relevant for low functioning ASD which appeared to be the majority of our cohort |
| CGI-I was added to the HSQ-ASD as a co-primary outcome (instead of being a secondary outcome) | June 2018  4 months before any statistical analyses were conducted | It became clear that in our cohort of low-functioning children, many participants had 4 or more items which were not applicable on the HSQ-ASD, invalidating the questionnaire. |

*Changes that were made after first registration of the study (as detailed in <https://clinicaltrials.gov/ct2/history/NCT02956226>) prior to study onset: When initially registered (Nov 2016) we planned on 3 co-primary outcome measures (HSQ, CBCL, APSI), 2 secondary measures (CGI-I, SRS) and safety. Prior to study onset, we changed to a single primary outcome (HSQ), with all others secondary. This change was updated in the Clinicaltrials.gov website a few days after recruitment onset (February 2017).

**Appendix 2: Anchoring instructions for rating the CGI-S and CGI-I – Behavioral Problems - ASD**

**Clinical Global Impression – Severity (CGI-S) - Behavioral problems- ASD**

*Severity of Behavioral difficulties during the past six months*

Please describe the four behavioral problems that are most disturbing for the participant, the family and the caregivers. Please state the frequency and severity of the problems in the last six months, and the impact on the participant, the family and the caregivers.

1. _______________________________________________________________________________________________________________________________________________________________________________________________________________________________________________________________________________________________________________________________________________________________________________________________________________________________________________________________________________________________________________________________________________________________________________________
2. __________________________________________________________________________________________________________________________________________________________________________________________________________________________________________________________________________________________________________________________________________________________________________________________
3. ____________________________________________________________________________________________________________________________________________________________________________________________________________________________________________________________
4. ____________________________________________________________________________________________________________________________________________________________________________________________________________________________________________________________

Considering your total clinical experience with this particular population, how severe are the behavioral problems at this time?

Please consider the above list and the following anchoring instructions and table.

0 = Not assessed 4 = Moderately ill

1 = Normal, not at all ill 5 = Markedly ill

2 = Borderline mentally ill 6 = Severely ill

3 = Mildly ill 7 = Among the most extremely ill patients

**7 – Dangerous - at least once a week:**

Injures self and / or others on purpose, in a manner that endangers health and at least once in the last 6 months required medical treatment. The violent outbursts occur at least once a week.

**6 - Extremely anxious** **(on a daily basis)** *or:*

**Requires restraining at least once a week** *or:*

**Dangerous - at least once a month**

Every day: Is extremely anxious or nervous, cries or screams for long periods (30 minutes), constantly asks repeated questions, severe restlessness.

Or:

At least once a week: Injures self or others or breaks objects, in a manner that requires physical restraint. The outbursts last longer than 10 minutes.

or:

At least once a month (but less than once a week): Injures self and / or others on purpose, in a manner that endangers their health and at least once in the last 6 months required medical treatment; engages in unwanted sexual behavior (unwanted comments or physical contact, exposing himself/ herself, masturbating in public)

**5 – Marked irritability (on a daily basis)** *or:*

**Prominent outbursts - at least once a week** *or:*

**Requires restraining at least once a month**

Every day: Is very irritable or moody, soils self, eats non-food items, engages in compulsive behavior for long periods; Get fixated on a person in a way that is very annoying or intrusive to that person.

or:

At least once a week: Outbursts that include physical or verbal violence or breaking objects and their length or severity does not require physical restraint; Takes off from home or work without regards for safety; Threatens to hurt or kill someone

or:

At least once a month (but less than once a week): Injures self or others or breaks objects, in a manner that requires physical restraint for more than 10 minutes.

**4 – Stubborn (on a daily basis)** *or:*

**Oppositional defiant behavior at least once a week** *or***:**

**Prominent outbursts at least once a month**

Every day: is stubborn; moody; lies, cheats or steals; demands must be met immediately; stamps feet or bangs objects or slams doors; sleep problems that disturb others from sleeping

or:

At least once a week: refuses to participate in daily activities or to perform daily tasks, in a way that causes significant difficulty.

At least one outburst involving physical or verbal violence or breaking objects that did not require physical restraint in the last 3 months and at least one more in the preceding 3 months.

or:

At least one outburst requiring physical restraint in the past six months.

**3 – Argues or Needy**

Every day: overly needy or dependent; eating problems; stubbornness and recalcitrance that does not involve a significant difficulty in day-to-day management.

or

At least once a month (but less than once a week): refuses to participate in daily activities or to perform daily tasks, in a way that causes significant difficulty.

or:

At least one prominent outburst (that included physical or verbal violence or breaking objects) in the past six months

**2. Any other behavioral difficulties**

**1 - No behavioral difficulties**

**CGI-S rating of Behavioral Problems in the last 6 months.**

**Please circle all that apply. The highest rating should be given based on the table**

| **Rating** | **Stubbornness^5^** | **Oppositional defiant^4^** | **Prominent outbursts^3^** | **Requires restraining^2^** | **Dangerous^1^** |
| --- | --- | --- | --- | --- | --- |
| 7 |  |  |  |  | At least once a week |
| 6 |  |  |  |  | At least once a month |
| 6 |  |  |  | At least once a week |  |
| 5 |  |  |  |  | At least once in last six months |
| 5 |  |  |  | At least once a month |  |
| 5 |  |  | At least once a week |  |  |
| 4 |  |  |  | At least once in last six months |  |
| 4 |  |  | At least once in last 3 months |  |  |
| 4 |  | At least once a week |  |  |  |
| 3 |  |  | At least once in last six months |  |  |
| 3 |  | At least once a month |  |  |  |
| 3 | At least once a week |  |  |  |  |
| 2 |  | At least once in last six months |  |  |  |
| 2 | At least once a month |  |  |  |  |
| 1 | Less than once a month |  |  |  |  |

^1^ **Dangerous** - Injures self and / or others on purpose, in a manner that endangers health and at least once in the last 6 months required medical treatment.

^2^ **Requires restraining** - Patients who injure themselves or others or break objects, in a manner that requires physical restraint. The outbursts last longer than 10 minutes

^3^ **Prominent outbursts** - Outbursts that include physical or verbal violence or breaking objects and their length or severity does not require physical restraint for more than 10 minutes

^4^ **Oppositional defiant** - Refusal to participate in daily activities or to perform daily tasks, in a way that causes significant difficulty in day-to-day management

^5^ **Stubbornness** - Stubbornness and recalcitrance that does not involve a significant difficulty in day-to-day management

**Clinical Global Impression – Improvement (CGI-I) - Behavioral problems- ASD**

Developed in consultation with Dr. Elizabeth Berry-Kravis

Clinicians should rate the total change, across all behavioral symptoms due to ASD, whether or not related to the experimental drug treatment.

Functional impairment from maladaptive behaviors such as anxiety, hyperactivity, impulsivity, short attention span, aggression, tantrums, and self-injurious behavior, should be considered in the rating, Changes in the HSQ-ASD and the APSI scores can be considered if these are obtained at the visit when the CGI-I is being done.

Please consider the CGI-S form (completed at baseline) and the following anchoring instructions.

0 = Not assessed                            4 = No Change

1 = Very Much Improved              5 = Minimally Worse

2 = Much Improved                      6 = Much Worse

3 = Minimal Improvement            7 = Very Much Worse

1.   **Very Much Improved.**

This rating indicates a very substantial improvement in the participant’s overall behavior compared to baseline. Extensive improvement in the behavioral difficulties described at baseline is evident at home, school, public, and unfamiliar settings. There is no emergence or significant worsening of behavioral difficulties that were not listed at baseline. The improvement in behavior enables greater participation in many settings (e.g., home, school, indoor, and outdoor recreational activities) and improves the quality of life of the participant and the participant’s family. The improvement is sufficiently adequate so that there is no need for additional pharmacological or behavioral treatments to address behavioral difficulties. This degree of improvement would typically result in an improvement in the rating of the CGI-S behavioral problems -ASD compared with the baseline rating. A rating of 1 in the CGI-I behavioral problems -ASD might be given even if the participant still has some functional impairment due to behavioral problems as long as the improvement from baseline is very substantial.

2.   **Much Improved**.

This rating indicates a substantial improvement in the participant’s overall behavior compared to baseline. There is a clear-cut improvement in most of the behavioral difficulties described at baseline. The improvement is evident in several settings (for example home and school but not necessarily public or unfamiliar settings). This improvement substantially outweighs any emergence or worsening of other behavioral difficulties. The overall improvement in behavior enables higher participation in several settings (e.g., home, school, indoor or outdoor recreational activities) and improves the quality of life of the participant and the participant’s family. Despite the clear-cut improvement in behavior, there is still room for improvement. Additional pharmacological or behavioral treatments to address the behavioral difficulties may be considered.  This degree of improvement may (but not necessarily) result in an improvement in the rating of the CGI-S behavioral problems -ASD compared with the baseline rating.

3.   **Minimal Improvement**.

This rating indicates a modest improvement in the participant’s overall behavior compared to baseline. There is a clear improvement in one or more of the behavioral difficulties described at baseline. The improvement must be consistent and it should exceed occasional fluctuations in behavior.  The improvement can be confined to one setting but it should increase participation and/or quality of life in that setting. This improvement should outweigh any emergence or worsening of other behavioral difficulties. Despite the modest overall improvement in behavior, additional pharmacological or behavioral treatments to address the behavioral difficulties are usually necessary. This degree of improvement does not result in an improvement in the CGI-S behavioral problems -ASD rating.

4.   **No Change**.

This rating indicates no clear change or only negligible change in the participant’s overall behavior compared to baseline. There is no significant improvement in the behavioral difficulties described at baseline. Alternatively, there is an improvement in some behaviors but it does not outweigh worsening of other behavioral difficulties.  This rating also reflects minimal changes to the overall behavior that have no impact on participation or quality of life. Additional pharmacological or behavioral treatments to address behavioral difficulties are usually required. This degree of improvement does not result in any change in the CGI-S behavioral problems -ASD rating.

5.   **Minimally Worse**.

This rating indicates a modest worsening in the participant’s overall behavior compared to baseline. The worsening must be consistent and exceed occasional fluctuations in behavior.  The worsening can be confined to one setting but it should decrease participation and/or quality of life in that setting. Additional pharmacological or behavioral treatments are required to address the worsening in behavior. This degree of worsening does not result in a worse CGI-S behavioral problem -ASD rating compared to baseline.

6.   **Much Worse**.

This rating reflects a substantial worsening in the participant’s overall behavior compared to baseline. The worsening is evident in several settings (e.g., home, school, public or unfamiliar settings) and decreases participation in those settings. The worsening in behavior leads to a decrease in the quality of life of the participant and the participant’s family. There is a need for more support and additional pharmacological or behavioral treatments to address the worsening in behavior. This degree of worsening might (but not necessarily) result in a worsening in the rating of the CGI-S behavioral problems -ASD compared with the baseline rating.

7.   **Very Much Worse**.

This rating indicates a very substantial worsening in the participant’s overall behavior compared to baseline. Extensive worsening in the overall behavior is evident in multiple settings. The worsening in behavior leads to substantially lower participation in many settings (e.g., home, school, indoor or outdoor recreational activities) and considerably decreases the quality of life of the participant and the participant’s family. There is a need for more support and additional pharmacological and behavioral treatments to address the worsening of behavioral difficulties. This degree of worsening would typically result in a worsening in the rating of the CGI-S behavioral problems -ASD compared with the baseline rating.

**Additional file 1: Table S1**. Serious adverse events reported during 12-week treatment periods.

| Whole-plant extract  N= 95 | 1 – had a seizure and was admitted to the emergency room (ER)  1 – had a questionable suicidal attempt (took a knife from the kitchen, didn’t harm himself. His psychiatrist didn’t consider it a suicidal attempt and no treatment change was made.) |
| --- | --- |
| Pure cannabinoids  N= 93 | 2 – had a seizure and were admitted to the ER  1 – broke his leg and was admitted to the ER |
| Placebo  N= 9**4** | 1 - died unexpectedly due to rupture of brainstem arteriovenous malformation (never having received cannabinoids) |

**Additional file 1: Table S2A:** Adverse events (AEs) reported during 12-week periods and correlations with age, sex, concomitant medications and treatment dose. AEs reported in equal or higher frequency during placebo treatment are listed in *Supplemental Table 6B*.

| Correlation with use of antipsychotics    r_φ_ (p)^2^ | Correlation with use of medications – any  r_φ_ (p)^2^ | Correlation with dose of cannabinoids  in mg/kg/d  r_rb_ (p)^2^ | Correlation with sex^1^    r_φ_ (p)^2^ | Correlation with age  r_rb_ (p)^2^ | Placebo  N= 94 | Pure cannabinoids  N= 93 | Whole plant extract  N= 95 |  |
| --- | --- | --- | --- | --- | --- | --- | --- | --- |
|  |  |  |  |  | **7.5** | **24%** | **27%** | **Somnolence** |
| 0.06 (0.27) | **0.184** | **-0.126** | **0.145** | **0.126** | 7.5% | 18.5% | 20% | Mild |
|  | **(0.001)** | **(0.036)** | **(0.012)** | **(0.029)** | 0% | 5.5% | 7% | Moderate |
|  |  |  |  |  | **15%** | **21.5%** | **24%** | **Decreased appetite** |
| 0.01 (0.84) | 0.005 | 0.001 | 0.156 | 0.02 | 13% | 16.5% | 21% | Mild |
|  | (0.94) | (0.98) | (0.067) | (0.73) | 2% | 5.5% | 3% | Moderate |
|  |  |  |  |  | **4%** | **13%** | **12%** | **Weight loss** |
| -0.03 (0.66) | -0.004 | -0.113 | 0.10 | 0.08 | 3% | 12% | 9% | Mild |
|  | (0.94) | (0.059) | (0.09) | (0.17) | 1% | 1% | 3% | Moderate |
|  |  |  |  |  | **19%** | **34%** | **25%** | **Tiredness** |
| -0.02 (0.69) | 0 (1) | -0.02 (0.73) | 0 (1) | -0.02 | 18% | 28.5% | 21% | Mild |
|  |  |  |  | (0.74) | 1% | 5.5% | 4% | Moderate |
|  |  |  |  |  | **13%** | **19%** | **20%** | **Euphoria** |
| 0.09 (0.12) | 0.02 | **-0.118** | 0.04 | -0.07 | 12% | 16% | 15% | Mild |
|  | (0.73) | **(0.049)** | (0.44) | (0.24) | 1% | 3% | 5% | Moderate |
|  |  |  |  |  | **3%** | **14%** | **4%** | **Depression** |
| 0.005 (0.93) | 0.02 | **-0.169** | -0.03 | 0.07 | 3% | 12% | 3% | Mild |
|  | (0.76) | **0.005** | (0.56) | (0.24) | 0% | 2% | 1% | Moderate |
|  |  |  |  |  | **14%** | **27%** | **20%** | **Anxiety** |
| -0.05 (0.41) | 0.02 | -0.05 (0.39) | -0.07 | 0.08 | 11% | 25% | 17% | Mild |
|  | (0.75) |  | (0.21) | (0.14) | 3% | 2% | 3% | Moderate |
|  | -0.02 |  |  | 0.03 | **4%** | **18%** | **5%** | **Dry mouth** |
| -0.001 (0.99) | (0.74) | -0.10 (0.09) | 0.05(0.36) | (0.63) | 4% | 18% | 5% | Mild |
|  |  |  |  |  | **3%** | **6%** | **6%** | **Seizures** |
|  |  |  |  |  | 3% | 2% | 3% | Mild |
| -0.09 (0.10) | 0.04 | -0.07 (0.22) | **0.115** | 0.06 | 0% | 2% | 2% | Moderate |
|  | (0.48) |  | **(0.047)** | (0.27) | 0% | 2% | 1% | Severe |

^1^ Positive correlation means more prevalent in girls.

^2^ Phi coefficient (r_φ_) was used for binary variables (sex, use of medications); rank biserial correlation (r_rb_) was used for continuous variables (age, cannabinoid dose).

Somnolence was more prevalent in older ages, in girls and in those who used other medications.

Negative correlations with cannabinoid dose, probably resulted from decreasing the dose in those who had adverse events.

**Additional file 1: Table S2B:** Adverse events reported in equal or *higher* frequency during the 12- week placebo treatment compared to 12-week cannabinoid treatments.

| Placebo  N= 94 | Pure cannabinoids  N= 93 | Whole plant extract  N= 95 |  | P-value^ | Placebo  N= 94 | Pure cannabinoids  N= 93 | Whole plant extract  N= 95 |  |
| --- | --- | --- | --- | --- | --- | --- | --- | --- |
| **5%** | **6%** | **6%** | **Memory problems** |  | **18%** | **19%** | **17%** | **Motivation problems** |
| 5% | 6% | 6% | Mild |  | 18% | 19% | 17% | Mild |
| **30%** | **33%** | **28%** | **Aggression** |  | **26%** | **27%** | **26%** | **Mood Swings** |
| 21% | 20% | 18% | Mild |  | 25% | 24% | 23% | Mild |
| 9% | 13% | 10.5% | Moderate |  | 1% | 3% | 3% | Moderate |
| **2%** | **4%** | **5%** | **Tremor** |  | **11%** | **11%** | **8%** | **Disorientation** |
| 2% | 4% | 5% | Mild |  | 11% | 11% | 8% | Mild |
| **28%** | **21%** | **24%** | **Nervousness** |  | **14%** | **19%** | **15%** | **Unsteadiness** |
| 26% | 18% | 21% | Mild |  | 14% | 19% | 14% | Mild |
| 2% | 3% | 3% | Moderate |  | 0% | 0% | 1% | Moderate |
| **28%** | **28%** | **28%** | **Restlessness** |  | **4%** | **3%** | **7%** | **Dizziness** |
| 17% | 17% | 21% | Mild |  | 3% | 2% | 7% | Mild |
| 11% | 11% | 7% | Moderate |  | 1% | 1% | 0% | Moderate |
| **16%** | **15%** | **11%** | **Diarrhea** |  | **4%** | **7.5%** | **6%** | **Weakness** |
| 13% | 14% | 11% | Mild |  | 4% | 7.5% | 5% | Mild |
| 3% | 1% | 0% | Moderate |  | 0% | 0% | 1% | Moderate |
| **2%** | **1%** | **2%** | **Hair loss** |  | **3%** | **2%** | **2%** | **Tics** |
| 2% | 1% | 2% | Mild |  | 3% | 2% | 2% | Mild |
| **18%** | **16%** | **19%** | **Concentration problems** |  | **23%** | **16%** | **23%** | **Gain of appetite** |
| 17% | 13% | 18% | Mild |  | 21% | 15% | 21% | Mild |
| 1% | 3% | 1% | Moderate |  | 2% | 1% | 2% | Moderate |
| **12%** | **7.5%** | **7%** | **Headache** |  | **8.5%** | **9%** | **11.5%** | **Skin problems** |
| 10% | 5% | 7% | Mild |  | 7.5% | 8% | 11.5% | Mild |
| 2% | 2% | 0% | Moderate |  | 1% | 1% | 0% | Moderate |
| **3%** | **3%** | **3%** | **Suicidal thoughts** |  | **13%** | **11%** | **17%** | **Upset stomach** |
| 3% | 2% | 3% | Mild |  | 12% | 11% | 14% | Mild |
| 0% | 1% | 0% | Moderate |  | 1% | 0% | 3% | Moderate |
| **0%** | **1%** | **3%** | **Blurred vision** |  | **7.5%** | **10%** | **5%** | **Delusions** |
| 0% | 1% | 3% | Mild |  | 7.5% | 10% | 5% | Mild |
| **6%** | **6%** | **4%** | **Hallucinations** | **0.326** | **15%** | **10%** | **10.5%** | ***Weight gain*** |
| 6% | 5% | 3% | Mild |  | 13% | 10% | 10.5% | Mild |
| 0% | 1% | 1% | Moderate |  | 2% | 0% | 0% | Moderate |
|  |  |  |  | **0.106** | **36%** | **23%** | **29.5%** | ***Disturbed sleep*** |
|  |  |  |  |  | 27% | 12% | 17% | Mild |
|  |  |  |  |  | 10% | 11% | 13% | Moderate |
|  |  |  |  | **0.026** | **14%** | **5%** | **5%** | ***Confusion*** |
|  |  |  |  |  | 14% | 5% | 4% | Mild |
|  |  |  |  |  | 0% | 0% | 1% | Moderate |

**^** P-values appear only for adverse events reported in higher frequency during the placebo treatment. Please see Table 3 for adverse events reported in higher frequency during the cannabinoid treatments.

**Additional file 1: Table S3.** Change from baseline to end of treatment period 2 in total scores of HSQ-ASD, SRS-2 and APSI. ^*^

|  | Median (Range) [n] | | |
| --- | --- | --- | --- |
| **Assessment** | Placebo | Pure Cannabinoids | Whole Plant extract |
| **HSQ-ASD** | -0.1 (-2.5 to 2.7) [35] | -0.6 (-3.1 to 1.7) [40] | 0.0 (-3.2 to 4.7) [41] |
| **SRS-2** | -2.2 (-26 to 42) [30] | -9.3 (-43 to 22) [33] | -4.8 (-61 to 19) [33] |
| **APSI** | -0.42 (-14 to 14) [37] | -2.4 (-40 to 15) [39] | -0.72 (-19 to 32) [40] |

*Results of period 2 were not used to assess efficacy due to a possible carry-over effects and are presented only for transparency.

HSQ- Home Situations Questionnaire; SRS-2 - Social Responsiveness Scale, 2^nd^ edition; APSI - Autism Parenting Stress Index.

**Additional file 1: Table S4.** Impact of possible moderators of response to cannabinoids (per outcome, during period 1). Results are presented as Odds Ratios (p-Values) [95% confidence interval].

|  | **CGI-I**^1^ | **HSQ-ASD**^2^ | **APSI**^2^ | **SRS-2**^3^ |
| --- | --- | --- | --- | --- |
| Pure cannabinoids | 1.67 (0.335) | 1.47 (0.457) | 1.78 (0.269) | 1.83 (0.360) |
| Whole-plant extract | **3.56 (0.015)**  **[1.31, 10.28]** | 1.89 (0.211)  [0.7, 5.18] | 2.31 (0.108)  [0.84, 6.57] | **6.08 (0.003)**  **[1.9, 21.82]** |
| Sex (Female) | 0.44 (0.146)  [0.14,1.27] | **0.25 (0.027)^$^**  **[0.07, 0.81]** | 0.56 (0.270)  [0.19, 1.53] | **0.17 (0.039)^$^**  **[0.02, 0.75]** |
| Age | **0.87 (0.024)^#^**  **[0.77, 0.98]** | 0.92 (0.191)  [0.81, 1.04] | **0.9 (0.094)^#^**  **[0.79, 1.01]** | 1.07 (0.310)  [0.94, 1.24] |
| ADOS total score at baseline | 0.95 (0.145) | 0.97 (0.480) | 0.99 (0.769) | 0.96 (0.275) |
| Concomitant use of antipsychotics | 1.06 (0.888) | 0.78 (0.556) | 1.09 (0.842) | 0.52 (0.246) |
| Concomitant use of SSRIs | 1.42 (0.542) | 1.15 (0.804) | 0.97 (0.952) | 0.56 (0.386) |
| Concomitant use of antiepileptics | 3.14 (0.113) | 1.06 (0.942) | 2.97 (0.147) | 0.96 (0.973) |
| Concomitant use of stimulants | 0.92 (0.905) | 1.27 (0.728) | 1.51 (0.564) | 1.86 (0.409) |
| Reported somnolence | **3.51 (0.011)^^^**  **[1.36, 9.58]** | 0.5 (0.180)  [0.18, 1.37] | 1.24 (0.668)  [0.46, 3.38] | 0.54 (0.342)  [0.15, 1.85] |

Results of 4 separate multivariate logistic models, (one for each study outcome) are presented.

Treatment (Placebo as control), sex (Male as control), medications use and reported somnolence were entered into the logistic model as categorical parameters while Age and ADOS total score were entered as continuous parameters.

**^1^** very much improved or much improved; **^2^** At least a 25% decrease in post-treatment score; **^3^** At least a 15% decrease in post-treatment score.

**^$^**  Males were more likely to respond to cannabinoids on the HSQ-ASD and SRS-2 assessments.

**^#^** Younger children were more likely to respond to cannabinoids on the CGI-I and APSI assessments.

**^^^** Children who had somnolence during cannabinoid treatment were more likely to respond on the CGI-I.

SSRIs: Selective serotonin reuptake inhibitors.

**Additional file 1: Table S5.** Impact of cannabinoid dose (in mg/kg/d) on treatment response (per outcome, during period 1). Results are presented as Spearman's ρ (p-Values).

|  | **CGI-I**^1^ | **HSQ-ASD**^2^ | **APSI**^2^ | **SRS-2**^3^ |
| --- | --- | --- | --- | --- |
| Pure cannabinoids | 0.05 (0.74) | -0.10 (0.52) | -0.05 (0.73) | 0.03 (0.89) |
| Whole-plant extract | **-0.29 (0.0499)^#^** | 0.20 (0.22) | 0.06 (0.73) | 0.22 (0.21) |

Correlation between the treatment dose (per Kg of body weight) and treatment response were assessed using the Spearman's rank correlation coefficient.

The average treatment dose during the first period was 5.7 ± 2.6 mg/kg/d of CBD in the whole-plant extract arm and 5.9 ± 2.7 mg/kg/d of CBD in the pure cannabinoids arm (median 5.2 and 5.6 respectively). The average dose per kg is lower than the target dose (10mg/kg/d) as many participants weighted over 42 kg and reached the maximal daily dose and some had adverse events to higher dose.

**^#^**Higher dose of whole plant extract (per Kg of body weight) correlated with Higher improvement in the CGI-I (in this scale lower rate represent higher improvement).

**Additional file 1: Figure S1. Impact of cannabinoid treatment on disruptive behavior and core symptoms of ASD – Within-subjects analysis** (comparing responses to placebo and cannabinoids in participants who received both treatments). This analysis was originally planned but could not be used due to a treatment order effect (all treatments were more effective in Period 1).


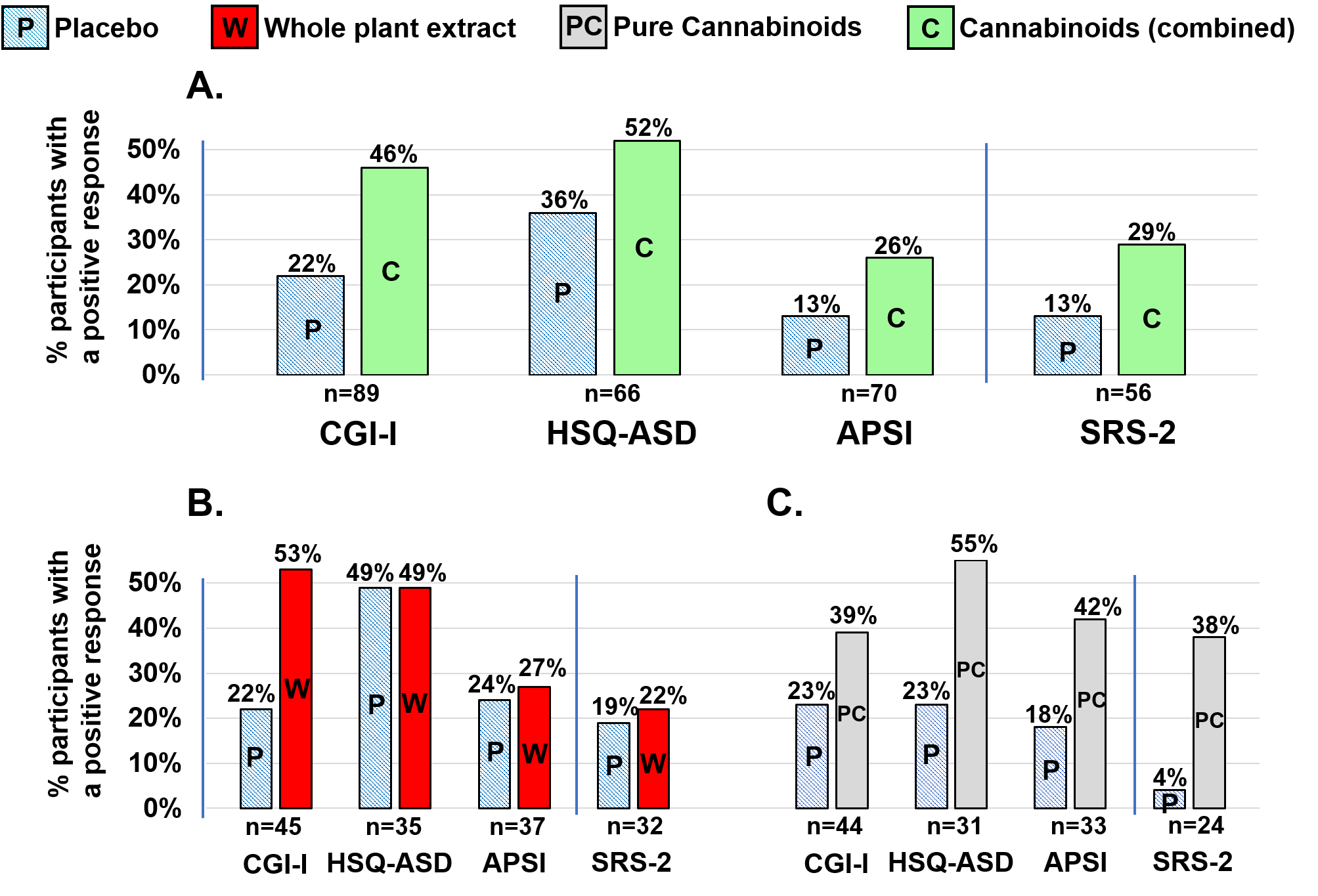


Response rates to 12-week treatment in participants who received both placebo and cannabinoid treatment (either whole-plant extract or pure cannabinoids) and had valid scores before and after both treatment periods, are presented in a within-subject analysis. Panel A: Cannabinoid treatment (either whole-plant extract or pure cannabinoids) versus placebo; Panel B: Whole-plant extract versus placebo; Panel C: Pure cannabinoids versus placebo.

Behavioral assessments: Clinical Global Impression-Improvement (CGI-I, a positive response was defined as a rating of 'much improved' or 'very much improved'); Home Situation Questionnaire-ASD (HSQ-ASD) and Autism Parenting Stress Index (APSI): positive response was defined as at least 25% decrease in post-treatment scores.

Core symptoms assessed per Social Responsiveness Scale – 2^nd^ edition (SRS-2). A positive response was defined as at least a 15% decrease in post-treatment score.

**Additional file 1: Figure S2. Impact of cannabinoid treatment and placebo on behavioral problems: comparison of first treatment period and second treatment period.** These results are presented only for transparency and were not used to assess efficacy.


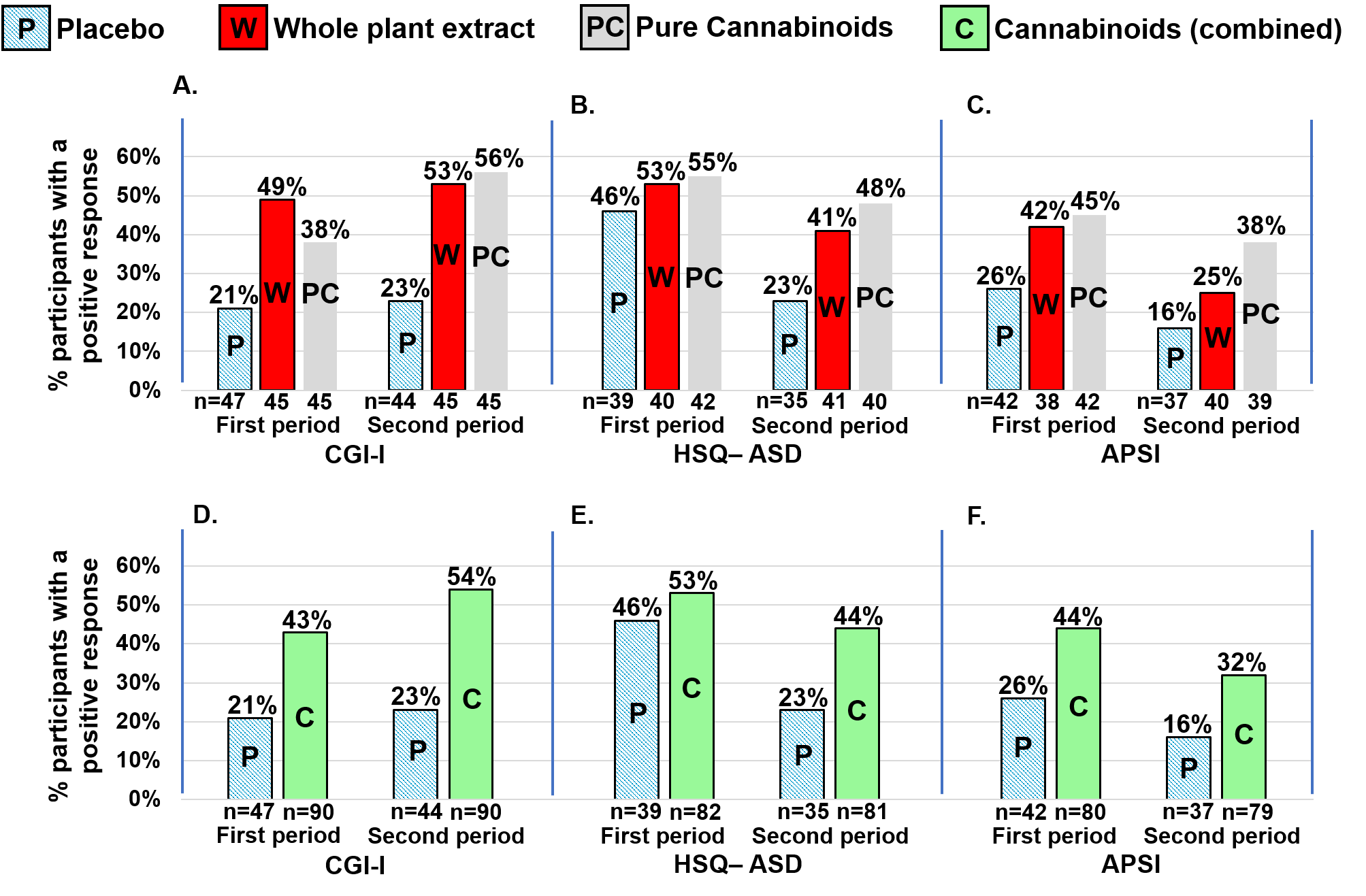
 Response rates to 12-week treatment in participants who had valid scores in pre- and post-treatment assessments. Response rates are presented for both treatment periods on 3 different behavioral assessments: A: Clinical Global Impression-Improvement (CGI-I, positive response defined as a rating of 'much improved' or 'very much improved'); B: Home Situation Questionnaire – ASD (HSQ-ASD, positive response defined as at least a 25% decrease in post-treatment score)^29^; C: Autism Parenting Stress Index (APSI, positive response defined as at least a 25% decrease in post-treatment score). D-F: Response rates to placebo and cannabinoids combined (either whole-plant extract or pure cannabinoids).

**Additional file 1: Figure S3. Impact of cannabinoid treatment on core symptoms of ASD as reflected by change in SRS-2 scores following treatment: comparison of first and second treatment periods**. These results are presented only for transparency and were not used to assess efficacy.


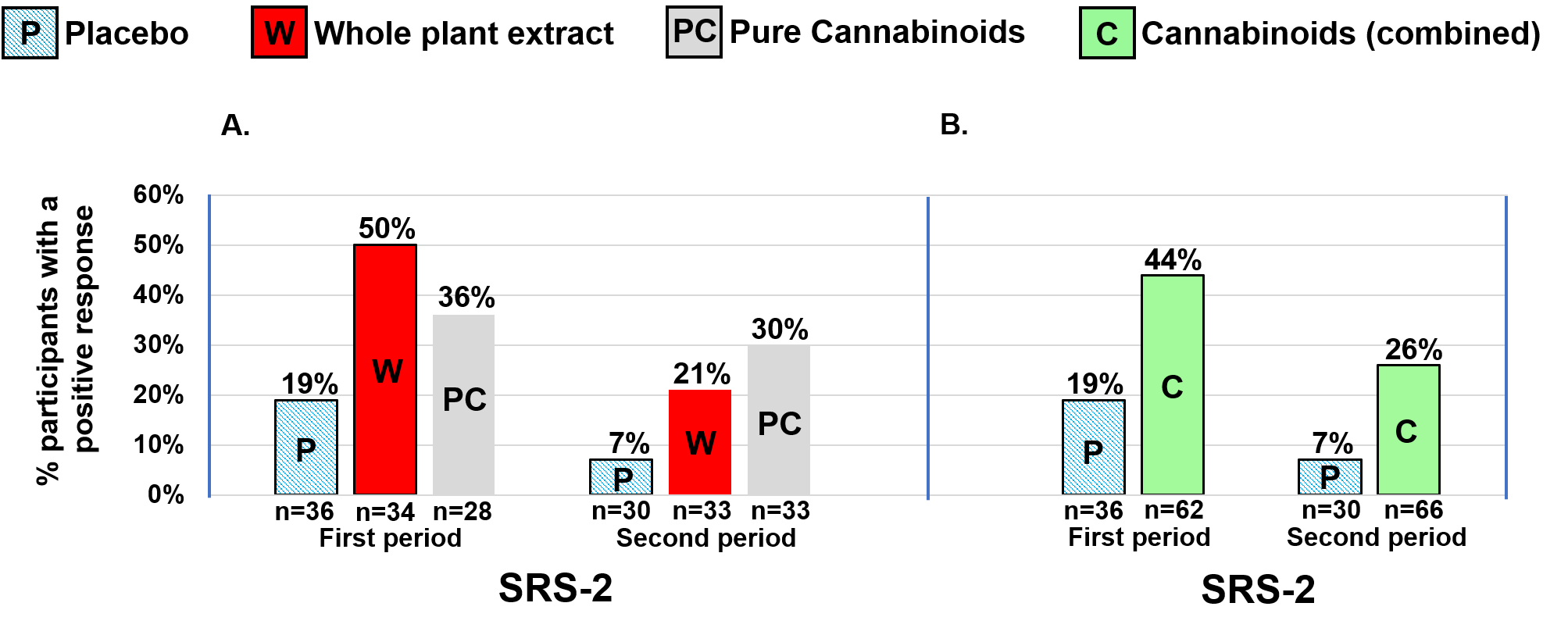


Response rates to 12-week treatments in participants who had valid scores on the Social Responsiveness Scale – 2^nd^ edition (SRS-2) in pre- and post-treatment assessments. Although only the first period was used to assess efficacy, response rates are presented for both treatment periods. A positive response was defined as at least a 15% decrease in post-treatment score. Panel A: Response rates to whole-plant extract, pure cannabinoids and placebo; Panel B: Response rates to cannabinoid treatments combined (either whole-plant extract or pure cannabinoids) versus placebo.
